# Differences in Social Decision-Making between Proposers and Responders during the Ultimatum Game: An EEG Study

**DOI:** 10.3389/fnint.2017.00013

**Published:** 2017-07-11

**Authors:** Sibylle K. Horat, Anne Prévot, Jonas Richiardi, François R. Herrmann, Grégoire Favre, Marco C. G. Merlo, Pascal Missonnier

**Affiliations:** ^1^Laboratory for Psychiatric Neuroscience and Psychotherapy, Department of Medicine, Faculty of Science, University of Fribourg Fribourg, Switzerland; ^2^School of Health Sciences (HEdS-FR), University of Applied Sciences and Arts Western Switzerland Fribourg, Switzerland; ^3^Laboratory of Neurology and Imaging of Cognition, Department of Neuroscience, University of Geneva Geneva, Switzerland; ^4^Department of Rehabilitation and Geriatrics, University Hospitals of Geneva Chêne-Bourg, Switzerland; ^5^Sector of Psychiatry and Psychotherapy for Adults, Mental Health Network Fribourg (RFSM) Marsens, Switzerland

**Keywords:** ultimatum game, social cognition, decision-making, event-related potentials, independent component analysis, brain source reconstruction

## Abstract

The Ultimatum Game (UG) is a typical paradigm to investigate social decision-making. Although the behavior of humans in this task is already well established, the underlying brain processes remain poorly understood. Previous investigations using event-related potentials (ERPs) revealed three major components related to cognitive processes in participants engaged in the responder condition, the early ERP component P2, the feedback-related negativity (FRN) and a late positive wave (late positive component, LPC). However, the comparison of the ERP waveforms between the responder and proposer conditions has never been studied. Therefore, to investigate condition-related electrophysiological changes, we applied the UG paradigm and compared parameters of the P2, LPC and FRN components in twenty healthy participants. For the responder condition, we found a significantly decreased amplitude and delayed latency for the P2 component, whereas the mean amplitudes of the LPC and FRN increased compared to the proposer condition. Additionally, the proposer condition elicited an early component consisting of a negative deflection around 190 ms, in the upward slope of the P2, probably as a result of early conflict-related processing. Using independent component analysis (ICA), we extracted one functional component time-locked to this deflection, and with source reconstruction (LAURA) we found the anterior cingulate cortex (ACC) as one of the underlying sources. Overall, our findings indicate that intensity and time-course of neuronal systems engaged in the decision-making processes diverge between both UG conditions, suggesting differential cognitive processes. Understanding the electrophysiological bases of decision-making and social interactions in controls could be useful to further detect which steps are impaired in psychiatric patients in their ability to attribute mental states (such as beliefs, intents, or desires) to oneself and others. This ability is called mentalizing (also known as theory of mind).

## Introduction

Social cognition examines processes involved during social interactions, such as understanding oneself and others, inferring somebody else’s thoughts, or identifying emotions and desires (Lieberman, 2007; Green et al., 2015). This process is an innate ability in humans known as the theory of mind, which allows normal social interactions. Deficits in this ability may lead to cognitive or developmental impairment, and have been reported to occur in some psychiatric diseases including schizophrenia or addiction to drugs or alcohol (Baron-Cohen et al., 1985; Uekermann and Daum, 2008; Ng et al., 2015).

Using experimental economic situations to study social interactions has proven to be particularly relevant as they are close to real-life social interactions. In this context, the Ultimatum Game (UG), a standard economic game, has gained importance in research on social cognition in healthy individuals. The UG is a simple tool that can be used to examine physiological correlates of decision-making processes. In this game, a “Proposer” owns a certain sum of money and is asked to propose a share of this money to a “Responder”. If the responder accepts the proposal, money is shared between both players according to the offer, whereas if he refuses both players end up with nothing (Guth et al., 1982). The goal is the same for both players, i.e., to gain the maximal amount of money.

To date, the behavioral aspect of the UG has been extensively described in humans (Guth et al., 1982; Fehr and Fischbacher, 2003). The neurobiological bases of this economic decision-making task are mainly being investigated using neuroimaging (Ritov, 1996; Zeelenberg et al., 1996; Sanfey et al., 2003; Bault et al., 2011). A functional magnetic resonance imaging (fMRI) study (Sanfey et al., 2003) and later investigations with repetitive transcranial magnetic stimulation (rTMS) and transcranial direct current stimulation (tDCS; Knoch and Fehr, 2007; Knoch et al., 2008) support the engagement of distinct brain areas in the decision-making process, combining emotional and cognitive areas of processing. However, the functional neuroimaging method lacks sufficient temporal resolution to explore brain processes occurring in the time scale of milliseconds. In contrast, electrophysiological approaches represent the most sensitive method to explore the timeline of early brain functions, and they are completely non-invasive as compared to rTMS. Therefore, we have chosen electroencephalography (EEG) as the appropriate method for our study.

Surprisingly, prior neuroimaging as well as electrophysiological studies (Boksem and De Cremer, 2010; Campanhã et al., 2011; Hewig et al., 2011; Qu et al., 2013) have focused on the responder condition only, whereas the proposer condition remains undiscussed for the most part. To our knowledge, there are no studies using the event-related potentials (ERPs) method to characterize ERP components specific to the proposer and responder conditions in control young adult players in the time range of 150–700 ms. The rare studies using ERPs to investigate the proposer condition focused on children and adolescents with or without cognitive deficits (see, Mesrobian et al., 2015; Soto-Icaza et al., 2015).

Previous investigations using ERPs revealed three major components related to cognitive processes in participants playing the UG. Studies primarily concentrated on changes of the Feedback-Related Negativity (FRN; also referred to as the medial frontal negativity), an ERP associated with feedback processing (Boksem and De Cremer, 2010; Wu et al., 2012; Alexopoulos et al., 2013; Qu et al., 2013; Ma et al., 2015). This component occurs in an extended latency range associated with the complexity of the stimulus (Massi and Luhmann, 2015), approximately 200–400 ms after stimulus onset (Massi and Luhmann, 2015; Sambrook and Goslin, 2015). Its amplitude increases whenever offers are judged as unfair (Osinsky et al., 2014; Kaltwasser et al., 2016). Interestingly, the FRN is also modulated by reward expectation. This component has been described to be maximal at fronto-central electrode sites (Wu and Zhou, 2009) and source reconstruction analysis localized the main generator in the dorsal region of the anterior cingulate cortex (ACC; Gehring and Willoughby, 2002; Miltner et al., 2003; Polezzi et al., 2010).

Secondly, a positive ERP component peaking around 200 ms (P2) after stimulus onset can also be seen in the early time course of the ERP. The P2 is usually observed maximally over frontal regions, and is modulated during cognitive tasks involving workload and attentional processes (Horat et al., 2016). Globally, the P2 is related to higher-order processes that involve the comparison of the eliciting event of the ERP with internal representation or expectation (Luck and Hillyard, 1994; Evans and Federmeier, 2007; Fiori et al., 2013). Interestingly, recent contributions pointed to an association of the P2 to an economic decision, with sources localized in the orbitofrontal cortex (Polezzi et al., 2010; Fiori et al., 2013).

Finally, a later positive ERP component associated with risk decision-making (Wu et al., 2011, 2012; Qu et al., 2013) is observed in the time window of 450–650 ms, referred to as late positive potential/component (LPP/LPC). This LPC is distributed over fronto-posterior electrode sites and is linked to the activation of sources within the insula, ventro-medial prefontal and posterior cingulate cortex (Liu et al., 2012). It is further associated with motivational relevance of the stimulus, and its amplitude increases proportionally to the engagement of high-level cognitive processes.

Despite the clear differences in the task conditions of responder and proposer, the final goal of gaining a maximal amount of money remains the same regardless of the condition. In this context, a shared electrophysiology but also differences between these two conditions should be expected. Characterizing both conditions in healthy adult controls is particularly important, especially since it has been reported that social cognition is impaired in schizophrenia and other psychiatric diseases. It strongly influences the daily functioning of these patients (Green et al., 2015; Healey et al., 2016), thus making it a critical field of psychiatric clinical research. The link between cognitive control deficits and dysfunctions of social cognition is well established in the literature (Etkin et al., 2013). However, it still remains unclear, which steps of the processing of cognitive control are defective and result in dysfunctions of social cognition. Deeper knowledge of the processes underlying social cognition could thus be of great utility for the psychosocial treatment of psychiatric patients (e.g., Mueser et al., 2013; Tan et al., 2016). This requires specifying the temporal sequence of processes involved in social decision-making, which are reflected by ERP components, in a healthy control population first.

The aim of the present study was thus to compare the two conditions in healthy young adult individuals, in order to analyze underlying brain processes and characterize potential differences and similarities. More precisely, we aimed to investigate whether the ERP parameters (amplitude and latency) of the P2, LPC and FRN differed according to the condition of the participant. Additionally, we unveiled a supplementary component around 190 ms (N2) in the proposer condition only. Therefore, in order to fully characterize this N2 we also performed: (1) an independent component analysis (ICA; for review see Makeig et al., 1997), which was used as an index of specific activation; and (2) an electrical source localization (LAURA; for review see Michel et al., 2004), which identified the activated regions. Characterizing both conditions in healthy controls could further allow using the UG as a tool to study impairments of the theory of mind in psychiatric patients.

## Materials and Methods

### Participants

Twenty healthy, French-speaking volunteers (10 males, 10 females; mean age 26.1 (± 4.2 SD) years, age-range 20–35) participated in the study.

All participants showed a normal cognitive functioning when tested with the extensive neuropsychological CogState Battery (see www.cogstate.com for details) including (Table 1): the Groton Maze Learning Test and Set-Shifting Task for executive functions; Detection Task for psychomotor function; Identification Task for visual attention; Groton Maze Learning Test delayed recall for visual learning and memory; International Shopping List Task and Delayed Recall for verbal memory; One Back Task for working memory; as well as the Social-Emotional Cognition Task for social cognition.

**Table 1:** CogState neuropsychological performances for participants (*n* = 20).

Tasks	Mean (SD)
**Executive Function**	
Set-Shifting Task—ER	16.05 (10.73)
**Executive Function/Spatial Problem Solving**	
Groton Maze Learning Test—ER tot	39.10 (8.08)
**Psychomotor Function/Speed of Processing**	
Detection Task speed, log_10_(ms)	2.48 (0.07)
**Visual Attention/Vigilance**	
Identification Task speed, log_10_(ms)	2.68 (0.04)
**Visual Learning and Memory**	
Groton Maze Learning Test—DR	4.45 (2.65)
**Verbal Learning and Memory**	
International Shopping List	
- CR tot	29.75 (2.47)
- DR	10.60 (1.28)
**Working Memory**	
One Back Task—AP	1.25 (0.17)
**Social Cognition**	
Social-Emotional Cognition Task—AP	1.18 (0.07)

*Notes: Data are presented as mean (SD). AP, accuracy of performance (arcsine transformation of the square root of the proportion of correct responses); ER tot, total number of errors; DR, delayed recall (number of correct responses); CR tot, total number of correct responses. See text for details*.

Participants all had normal or corrected-to-normal visual acuity and neither reported a history of sustained head injury, nor neurological or psychiatric disorders. Moreover, none exhibited a severe physical impairment or reported alcohol or drug abuse in questionnaires. Informed consent was obtained from all subjects, and all were naive to the UG. The study was approved by the Ethical Committee of the University of Fribourg (reference number: 054/13-CER-FR) and was conducted in line with the Helsinki Declaration.

### Task and Procedure

**Figure 1:**
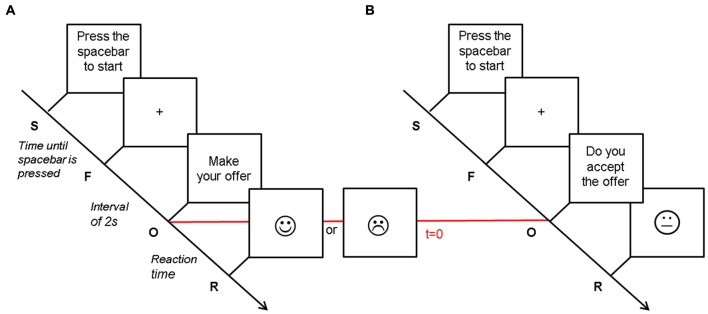
Illustration of the Ultimatum Game. Tasks differed in response requirement. Each trial began with the instruction to press the spacebar (S). As soon as participants did, a central fixation cross appeared in the middle of the monitor (F). After an interval of 2 s, participants saw a message (O) indicating to either make an offer (**A**—Proposer) or accept or reject an offer (**B**—Responder), depending on the condition. This time point was considered as our *t* = 0 for the ERP analyses, and is labeled with a horizontal red line. The response to the offer (R) was displayed simultaneously when participants pressed the button indicating their decision. It presented as a smiling or frowning face in the proposer condition, whereas the smiley was neutral in the responder condition. This figure has been adapted from Horat et al. (2017).

The UG is originally an anonymous, single-shot two-player game, in which the “Proposer” has a certain sum of money at his disposal and must propose a share of this money to the “Responder”, who can either accept or reject this offer. If the responder accepts the proposal, money is shared accordingly and both win money. However, if the responder refuses, both players end up with (Horat et al., 2017). Classically, the game ends after the responder’s decision (Guth et al., 1982). In the present version of the UG, each participant played both the role of the proposer (90 trials, Figure 1A) and of the responder (90 trials, Figure 1B) in three alternate blocks of 30 trials each (~180 s). In the first block of the protocol, the participant acted as the proposer; during the second block the other player (computer player) made the offer and the participant acted as the responder, and so on. Before each block, participants were informed about the nature of the task. Participants were also told to play the UG trying to maximize their gain as much as possible, and were instructed about the outcome of an “Accept” or “Reject” response. The overall experiment, including head cap installation, lasted about 70 min.

The UG was implemented using the E-Prime software (Psychology Software Tools, Inc., Sharpsburg, PA, USA) for stimuli presentation, trigger sending and response recording. The instructions of the task were provided in written form on a computer monitor. The task involved a take-it-or-leave-it split of 10 virtual Swiss Francs (CHF). Each trial began after the participant pressed on the spacebar followed by an interval of 2 s (preparatory period) during which participants were instructed to maintain their gaze on the central fixation cross on the computer monitor.

For each trial as a proposer, the participant was asked to pay attention to an introductive message (“Please, make your offer”) at the center of the monitor and press the number key corresponding to the digit in the range of [1,…,9] CHF that he intended to offer. The responder’s decision of accepting or rejecting the proposition was shown through a face diagram (a smiley) that either smiled or frowned (Figure 1A).

When the participant acted as responder, an offer in the range of [1,…,9] CHF appeared at the center of the screen. The participant had unlimited time to consider the offer and press a key (1 for “accept” and 0 for “reject”) to respond. As it has been shown that the proposer’s facial expression influences the responder’s willingness to accept an offer (Mussel et al., 2013), neither positive nor negative feedback was provided, instead the smiley was neutral (Figure 1B).

The second player was a computer program (computer player), but participants were not told explicitly (task instructions mentioned a “second player”). Previous studies (Sanfey et al., 2003; Radke et al., 2012) have shown that participants reject unfair offers made by human players at a higher rate than those made by computer players. Our approach of not mentioning the nature of the opponent was chosen to minimize this effect.

**Figure 2:**
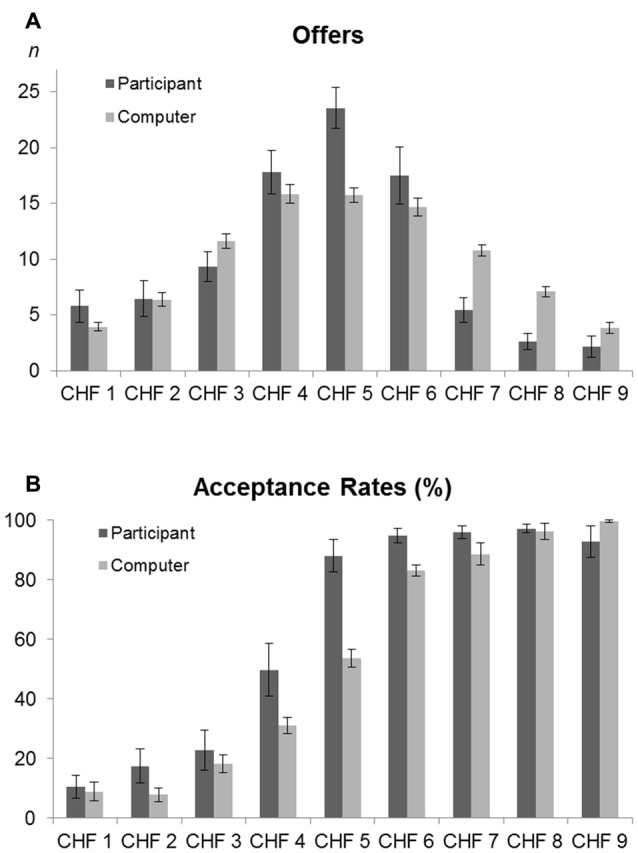
Offers and acceptance rates. **(A)** Number of offers made by the participants (dark gray—participants in proposer condition) and by the computer (light gray—participants in responder condition). The bars represent the amount of times every offer (1–9 CHF) was made, with standard errors (±SE). The distribution of the values (1–9 CHF) offered by the computer player was similar to the distribution of offers by the human proposer (*t* = −2.08; *p* = 0.06). **(B)** Acceptance rates of the participants (dark gray—participants in responder condition) and the computer (light gray—participants in proposer condition). The bars represent the percentage of accepted offers for each offered amount with standard errors (±SE). The distribution of the acceptance rates was not significantly different for the two players in both conditions (proposer: Friedman = 23.096; *p* = 0.151; responder: Friedman = 8.903, *p* = 0.446).

The computer program was implemented with offers according to a Gaussian probability curve over 5 (i.e., a replication of a human participant’s behavior), in order to simulate a human strategy (our computer algorithm was based on work from Roth et al., 1991; Slonim and Roth, 1998; Cameron, 1999). For example, when the human participant proposed one CHF or six CHF, the computer was programmed to accept 8% and 83% of the offers, hence reject 92% and 17%, respectively (Figure 2). The accuracy of the algorithm was confirmed by the performance results (Figure 2), which were similar for both computer and human players.

At the end of the session, no feedback on performance was provided. Electrophysiological and neuropsychological assessments were performed in the morning.

### Electrophysiological Recordings

**Figure 3:**
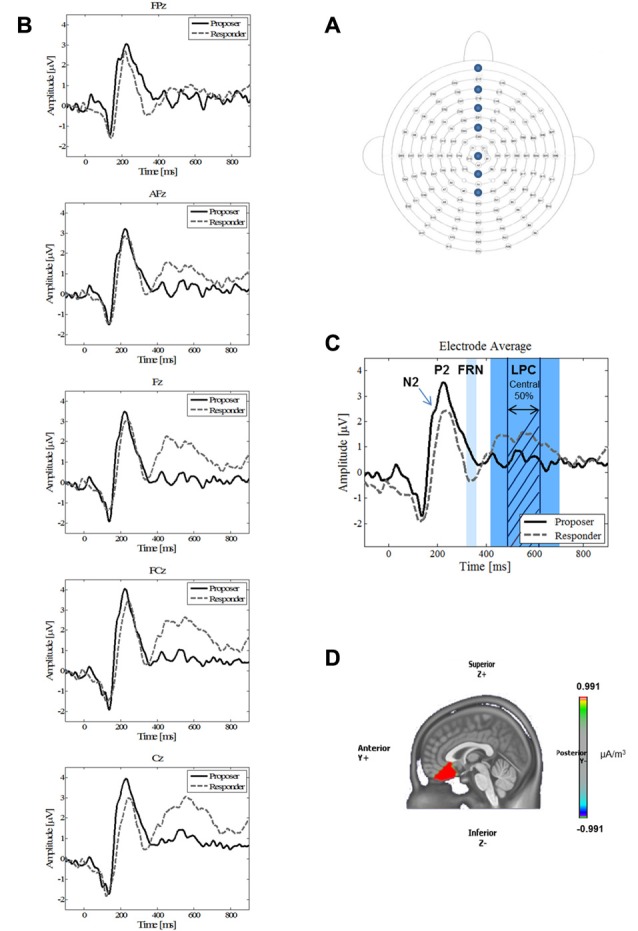
**(A)** Electrode positions. Placement of the 128 electrodes on the head cap. The electrodes relevant for this study are indicated, namely FPz, AFz, Fz, FCz, Cz, CPz and Pz from top to bottom (anterior to central head). **(B)** Grand average waveforms of five single electrodes. Grand average waveforms for proposer (solid black line) and responder (dashed gray line) during the Ultimatum Game (UG) for all outcomes (both acceptance and refusal of the offer) at the midline electrodes FPz, AFz, Fz, FCz and Cz. **(C)** Grand average waveform. Grand average waveform for the electrode average of five electrodes (FPz, AFz, Fz, FCz and Cz) of the proposer (solid black line) and responder (dashed gray line) conditions. The labels show the main components N2, P2, FRN and LPC. The band around the LPC component represents the complete length of the component, whereas the shaded area stands for the middle 50% that were taken for the analysis. In the responder condition, note the absence of a N2 component, the delayed latency and lowered amplitude for the P2, and the higher mean amplitude for the FRN and LPC. **(D)** Source reconstruction. Source reconstruction of the time-window of the N2 component (180–200 ms). A significant higher activation (*p* < 0.05) around the anterior cingulate cortex (ACC) was found for the proposer condition.

The parameters of the recording and data processing have been replicated from Horat et al. (2017). The participants were comfortably seated in an electrically shielded, sound- and light-attenuated room. The distance to the monitor was 1 m. Continuous EEG was recorded using 128 active surface Ag/AgCl electrodes (ActiveTwo MARK II Biosemi EEG System, BioSemi B.V., Amsterdam, Netherlands) mounted on a head cap (NeuroSpec Quick Cap; Figure 3A) and referenced to the common mode sense (CMS; active electrode). Linked right and left mastoid electrodes were used for a later re-referencing process. Additionally, right, left, supra-, and infra-orbital electrodes monitored horizontal and vertical eye movements. Electrode impedances were kept below 20 kΩ. Electrophysiological signals were sampled at 2048 Hz (DC amplifiers and software by Biosemi, USA). Markers corresponding to stimuli presentations and responses (proposer and responder offer types; Figure 1) were automatically documented with markers in the continuous EEG file. They were thereafter used off-line to segment the continuous EEG data into time-locked epochs.

### Data Processing

#### Electrophysiological Processing

The continuous EEG was referenced to mastoid channels using the Brain Vision Analyzer 2.0 software (Brain Products GmbH, Munich, Germany). EEG signals were corrected for blinks and eye movement artifacts through an ICA (Jung et al., 2000), which parameters were carefully selected for each individual to minimize any residual effects on the visually-inspected EEG signal. The total analysis window was 1200 ms, starting 200 ms before the appearance of the instruction to make a decision (i.e., stimulus onset: “make an offer” or “accept/reject the offer”; *t* = 0; Figure 1). Next, the EEG trials were automatically scanned for contamination by muscular or electrode artifacts (criteria for rejection: voltage step >70 μV/ms or peak to peak deflection within 200 ms intervals >200 μV/ms). The remaining trials were inspected visually to control for residual minor artifacts. Finally, the EEG data were analyzed with three different types of electrophysiological analyses: event-related potentials (ERPs), ICA and ERP source reconstruction analysis.

#### Event-Related Potentials Analysis

ERP analyses were performed by averaging the EEG signal over a window of 1200 ms with a 200 ms pre-stimulus onset period. The epochs were band-pass filtered between 0.3 Hz and 30 Hz (−48 dB/octave for a low-pass filter). ERPs were averaged with a 200 ms baseline epoch prior to stimulus onset.

The ERP components of interest were the P2 component, the FRN and the LPC. All of these components were identified in the grand-average waveform (implemented in BrainVision Analyzer 2.0 software, Brain Products GmbH, Munich, Germany).

To explore decision-making using ERPs, the P2 and FRN component analyses were restricted to anterior midline electrode locations (FPz, AFz, Fz, FCz, Cz) as these components are known to have a fronto-central maximum (Hewig et al., 2011; Yang and Zhang, 2011; Wu et al., 2012; Peterburs et al., 2013). On the other hand, since the LPC is reported to have a centro-parietal maximum (Itthipuripat et al., 2015), we took the measures of the electrode locations Fz, FCz, Cz, CPz and Pz.

For the P2, we measured the latency of the component from the stimulus onset to the time of the peak maximum, and its amplitude was measured at this peak maximum.

In the proposer condition, the FRN did not show an easily detectable peak and was slightly larger than in responders. Therefore, we measured the mean amplitude of the FRN based on the peak detected in responders ±10%, i.e., using the time window of 320–360 ms for both conditions.

Finally, as the LPC is a large component with no detectable peak in any condition, we measured the mean amplitude of the central 50% of the component, i.e., from 490 ms to 630 ms (Figure 3B). This method allowed a safe measurement of the LPC for all subjects in both conditions (Luck, 2005; Dickter and Kieffaber, 2014).

Furthermore, the inversion of polarity on the slope of the P2 component seen with ERP analysis strongly suggested the presence of an additional evoked activity around 190 ms (i.e., corresponding to a N2 component) in the proposer condition only. However, no proper peak could be identified accurately due to its low amplitude. Therefore, to fully characterize this evoked component, two complementary EEG methods were used: (1) an independent component activity (ICA) that confirms the presence of the component in the same time period as observed in the ERP; and (2) electrical source reconstruction (LAURA) that allows revealing the anatomical origin of this activity.

#### Independent Component Analysis (ICA)

ICA is a signal processing technique for blind source separation of a linear mixture of evoked electrophysiological data into temporally independent and spatially stationary sources (Makeig et al., 1997, 1999). We used the infomax ICA algorithm (Bell and Sejnowski, 1995; Amari et al., 1996) RUNICA, as implemented in EEGLAB with default values (including data sphering). The ICA software, EEGLAB, was provided by the Computational Neuroscience Laboratory of the Salk Institute (San Diego, CA, USA), and implemented in MATLAB version R2012b (Mathworks, Natick, MA, USA). The ICA algorithm was applied to ERPs recorded over all 128 electrodes for the two conditions, and we extracted 128 ICs. Since the ICA algorithm is initialized randomly, ICA was performed three times. Only reproducible ICs are reported in the present study. The ICA components were superimposed on the ERP waveform and those visually matching the ERP in the time period of interest (i.e., around the N2 component) were selected.

#### Electrical Source Localization (LAURA)

We estimated electrical sources underlying scalp-recorded data using a distributed linear inverse solution based on a local autoregressive average (LAURA) regularization approach (Grave de Peralta Menendez et al., 2004; also Michel et al., 2004 for a comparison of inverse solution methods). LAURA selects the source configuration that best mimics the biophysical behavior of electric fields (i.e., the activity at one point depends on the activity at neighboring points according to electromagnetic laws). The solution space is based on a realistic head model and includes 5010 solution points homogeneously distributed within the gray matter of the average brain of the Montreal Neurological Institute (courtesy of R. Grave-de Peralta Menendez and S. Gonzalez Andino, University Hospital of Geneva, Geneva, Switzerland). Intracranial source estimations were calculated for the time period of the N2 component (180–200 ms after stimulus onset), which was defined as a period of interest by the topographic analyses. In order to obtain those estimates, ERPs for each participant and each experimental condition were first averaged separately across the above-mentioned time period of interest to generate one time course per participant and experimental condition. The distribution of source activities across conditions was then statistically compared for each solution point using a paired *t*-test with a significance level of *p* < 0.05 uncorrected.

### Statistical Analysis

The normality of ERP data distribution was verified with the Shapiro-Francia test. As a log transform did not improve the normality of these parameters, native values were used. Electrode locations (FPz, AFz, Fz, FPz, Cz for P2 and FRN; and Fz, FPz, Cz, CPz and Pz for LPC) and task conditions (proposer, responder) were included as independent variables in a repeated-measure linear regression model to analyze their respective influence on each of the dependent variables (i.e., EEG measures). Statistical analyses were restricted to latency and amplitude of the P2 component for the individual average waveforms and mean amplitudes of the FRN and LPC. Statistical analyses were performed using the Stata software package, version 13.1. Corrected significance levels for multiple comparison testing were computed for the six statistical tests performed on each ERP component (4 values), and total gains and losses (2 values), using the Benjamini-Hochberg procedure (Benjamini and Hochberg, 1995) implemented with a spreadsheet developed by Dr. Manuel Weinkauf^1^. After correction, the statistical threshold for significance remained unchanged at *p* < 0.05.

## Results

### Behavioral Data

We compared the total gains of the participants in the proposer vs. the responder conditions. We found a mean overall gain of 202.45 ± 32.27 CHF when participants acted as proposer, whereas it was 352.75 ± 53.42 CHF when they acted as responder (*p* < 0.0001). The mean overall losses for participants were 270.75 ± 99.32 CHF and 96.2 ± 49.45 CHF for the proposer and responder conditions, respectively (*p* < 0.0001). Offer distribution and acceptance rates data are presented in Figure 2.

### Electrophysiological Data

#### Event-Related Potential Analysis

Analysis of averaged ERPs revealed the presence of the three ERP components previously described in the literature. Figures 3B,C present these waveforms in response to the two decision-making conditions, on a single electrode level (3B: FPz, AFz, Fz, FCz, Cz), as well as for the mean of these five electrodes (3C). The first component P2 is an early positive wave with a peak latency around 230 ms. The second component FRN is a negative wave around 320–360 ms. It was observed that the FRN occurred slightly later during the present study than is typically observed. We attributed this late arrival to a different complexity of our stimulus compared to other experiments (Massi and Luhmann, 2015). The third component is a large positive component lasting from 420 ms to 700 ms, which we identified as the LPC. Moreover, the ERPs revealed that, in the proposer condition only, participants elicited a negative component (N2) near 190 ms preceding the P2 component (Figure 3C).

As for the P2 parameters, both latency and amplitude were significantly delayed (*t* = 2.76, *p* = 0.013) and decreased (*t* = −3.36, *p* = 0.003), respectively, for the responder condition (see Table 2 for individual values of each electrode). In contrast, the FRN showed a more pronounced mean amplitude (*t* = −2.73, *p* = 0.013) in the responder condition (see Table 3 for individual values of each electrode). Finally, a significant difference of the mean amplitude of the LPC was observed, showing a higher amplitude for the responder condition (*t* = 3.47, *p* = 0.003; see Table 3 for individual values of each electrode). No electrode site effect or interactions between groups and electrode sites were observed.

**Table 2:** Amplitudes and latencies of the P2 component at five electrode sites in both task conditions.

	P2					
	Amplitudes (μV)			Latencies (ms)		
Electrode	Proposer	**	Responder	Proposer	*	Responder
	Mean (SD)		Mean (SD)	Mean (SD)		Mean (SD)
Mean	5.06 (3.05)		3.00 (1.75)	228.39 (24.11)		239.72 (20.52)
FPz	4.88 (3.39)		2.99 (1.92)	228.70 (23.25)		236.70 (23.39)
AFz	4.93 (2.73)		3.16 (1.62)	227.40 (23.84)		238.55 (19.69)
Fz	5.20 (2.94)		3.18 (1.65)	227.50 (23.06)		242.10 (19.85)
FCz	5.38 (3.11)		3.12 (1.72)	229.45 (24.47)		241.65 (18.94)
Cz	4.91 (3.08)		2.54 (1.85)	228.90 (25.91)		239.60 (20.74)

Notes: Data are presented as mean (SD). Amplitudes are in μV and latencies in ms. Significant global condition differences were found for all electrodes. **p < 0.01; *p < 0.05.

**Table 3:** Mean amplitude of the FRN (320–360 ms) and the LPC (490–630 ms) at five electrode sites in both task conditions.

FRN				LPC			
Mean Amplitude (μV)				Mean Amplitude (μV)			
Electrode	Proposer	*	Responder	Electrode	Proposer	**	Responder
	Mean (SD)		Mean (SD)		Mean (SD)		Mean (SD)
Mean	0.24 (1.75)		−0.78 (1.64)	Mean	−0.34 (1.04)		1.47 (1.13)
FPz	0.32 (1.83)		−0.91 (1.86)	Fz	−0.05 (0.93)		0.88 (1.05)
AFz	0.23 (1.75)		−0.65 (1.64)	FCz	−0.14 (0.99)		1.17 (1.11)
Fz	0.08 (1.62)		−0.72 (1.58)	Cz	−0.40 (1.09)		1.40 (1.08)
FCz	0.12 (1.66)		−0.81 (1.63)	CPz	−0.57 (1.03)		1.78 (1.18)
Cz	0.45 (1.90)		−0.82 (1.47)	Pz	−0.66 (1.15)		2.11 (1.20)

Notes: Data are presented as mean (SD). Mean amplitude is in μV. Significant global differences were found for all electrodes. **p < 0.01; *p < 0.05.

#### Independent Component Analysis (ICA)

**Figure 4:**
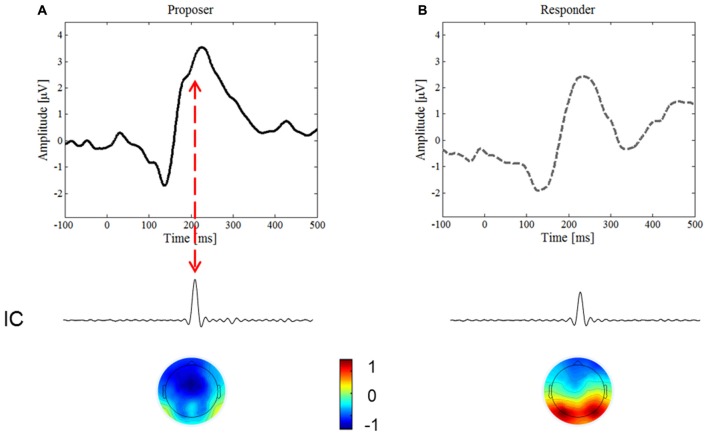
Independent Component Analysis (ICA). We focused on the IC predominately activated for the period of interest for the N2 component, around 180–200 ms after stimulus onset. **(A)** Represents the ERP waveform and the most important IC of the proposer condition, **(B)** of the responder condition, respectively. Note the lower variance of the IC for the responder than the proposer condition (2.2% and 5%, respectively), and the inversed topography of the brain map.

To endorse an underlying component of the N2 ERP, we conducted the ICA. An IC in the time period of the N2 was found in both conditions, yet only in the proposer condition was it time-locked with the inversed polarity peak visible on the upward slope of the ERP (Figure 4, red arrow), thus confirming the existence of this ERP component. The IC accounted for 5.0% of the variance in the proposer condition, but only for 2.2% of the variance in the responder condition.

The topographic distributions of the ICs are presented in Figure 4 below the corresponding IC. Note that the IC in the proposer condition is lateralized and of inversed polarity compared to the one of the responder, thereby further confirming the difference found in the proposer condition.

#### Electrical Source Imaging Localization (LAURA)

We conducted source imaging localization to identify the differences in the activity of the underlying brain regions of the N2 component, which was present only in the proposer condition. Figure 3D displays the grand mean source estimations for both experimental conditions over the 180–200 ms period. A significant difference between proposer and responder conditions (*p* < 0.05, uncorrected) was detected in a frontal cluster centered around the ACC, with a higher activation for the proposer condition.

## Discussion

The present study contributes to recent investigations on the electrophysiological bases of social cognition and decision-making in the context of the UG. Using ERPs, ICA and source reconstruction, we demonstrated that the time-course and the intensity of brain processing events involved in social decision-making are sensitive to the task condition (responder vs. proposer), even when the goal of earning a maximal amount of money is the same for both conditions.

As hypothesized, we found differences in the parameters of the ERP components, namely P2, LPC and FRN. Interestingly, our study is the first to reveal the presence of a negative polarity deflection in the upward slope of the P2, which was observed only in the proposer condition. This ERP appeared to correspond to the N2 as its characteristics, i.e., polarity, topography and latency, all closely coincide to those previously reported for the N2 (Kanske and Kotz, 2011). Our identification of an IC in the time range of 190 ms strongly supports the hypothesis of activation of the N2 component in the UG. The increase of IC activity in the proposer could explain why the N2 component was only visible in the waveform of the proposer condition.

### Conflict Resolution-Related Components

We clearly distinguished a FRN in both UG conditions at a fronto-central location around 320–360 ms after stimulus onset. The FRN mean amplitude was more pronounced in responders as compared to proposers.

Rule-based classifications for decision-making, including hypothesis generation and testing, are shared by both proposer and responder conditions and therefore cannot account for this discrepancy. Instead, the higher FRN amplitude indicates that the judgment of fairness was more pronounced for responders, thereby inducing a higher conflict between the competing responses to accept or reject the offer. In line with these observations, Sanfey and Chang (2008) showed that unfair offers induced a conflict between deliberative (accept every offer) and affective (reject the offer to punish the proposer for his unfair offer) motives in the responder condition. It is already well established in the decision-making literature (Boksem and De Cremer, 2010; Hewig et al., 2011; Alexopoulos et al., 2013; Qu et al., 2013) that the FRN component is strongly modulated by the participant’s judgment of fairness in the responder condition, which reinforces our findings.

The human player is involved in a conflict in both UG conditions. The decision of accepting or rejecting an offer seems to depend mainly on the judgment of fairness of the proposition in the responder condition, while participants had to choose a specific value for the next trial whilst considering the precedent computer response (i.e., accepted or rejected offer) in the proposer condition. Therefore, the proposer condition required the player to reevaluate his decision rule in order to optimize the gains following an unpredictable outcome. These multiple choices induce a great conflict and we suggest here that these conflict-related resolution operations were reflected in the supplementary N2 component that was visible in the proposer condition only. This interpretation is supported by literature describing that the N2 is sensitive to tasks involving high levels of conflict between competing responses (Yeung et al., 2004; Mennes et al., 2008; Yeung and Nieuwenhuis, 2009). The smaller IC activity in the responder condition suggests that the conflict of choosing a response is also present; yet, as there are only two options (accept/reject) compared to the nine options (each value) in the proposer condition, this conflict is smaller and, therefore, there is no visible N2 component in the ERP waveform of the responder.

Furthermore, our source reconstruction demonstrated a significantly higher activation of the ACC for the proposer condition in the N2 time-range. In the UG, the proposer must constantly reevaluate the decision rule in order to optimize the next gain, which means that he takes more risks than the responder. It has been demonstrated that the ACC is sensitive to the intensity of conflicts (Liston et al., 2006; Hsieh and Wu, 2011), resulting in a higher activity when conflicts are introduced (Schroder et al., 2012) and when stimulus–response rules are reversed (Randall and Smith, 2011), as is the case after the acceptance or rejection by the computer player of the offer made by the participant. Therefore, the greater ACC activity seen in our study supports an earlier involvement of conflict resolution processes of the proposer compared to the responder.

Taken together, the findings of the N2 in the proposer condition and the more pronounced amplitude of the FRN in the responder condition reveal that major processes such as risk-taking and conflict resolution involved in an economic decision occur at different times, depending on whether the participant acts as proposer or responder. Indeed, conflict resolution occurs early in the decision-making process in the proposer (~190 ms), whilst the resolution of the conflict to judge fairness occurs later (~330 ms) in the responder, leading to the increased FRN.

### Cognitive-Related ERP Components

Taking into account that models of decision-making involve a subtle combination of affective and cognitive motives (Sanfey and Chang, 2008), we also expected a modulation of the cognitive-based processes according to the task. In agreement with this hypothesis, we found a diminished amplitude of the P2, as well as a higher mean amplitude of the LPC for the responder condition.

The diminished amplitude of the P2 in the responder condition is due to a lower engagement of attention. It has been established that the attention level is related to mental effort (Miller et al., 2011), and we previously reported that a reduced allocation of attention results in a reduced P2 amplitude (Horat et al., 2016). Interestingly, in the UG the different number of options (nine in the proposer condition vs. two in the responder) does not require the same level of mental effort. The P2 could thus be modulated accordingly. In line with this idea, the responder condition requires less attention compared to the proposer. From a neurophysiological perspective, the amplitude of the ERP is linearly related to the number of neurons activated by the task (Niedermeyer, 1987). This physical property supports a strong relation between attention and characteristics of ERP parameters. Here, the lower mental effort requires less attention to the incoming stimulus in the responder and therefore results in a lower P2 amplitude as well as a delayed latency.

Meanwhile, the higher mean amplitude of the LPC component in the responder condition suggests the engagement of supplementary mental resources. It has already been suggested that the LPC may have functions in social evaluation (Wu et al., 2011). The fact that responders have the final say in the UG may increase their motivated attention, and therefore result in the higher LPC amplitude, as previously reported by van Hooff et al. (2011). Supporting this hypothesis, participants must compare the actual offer to a threshold they set for themselves, in order to decide whether to accept (if the offer is higher than their personal threshold) or reject (if the offer is lower) the sum proposed. Our findings support prior research which showed that the LPC is involved both in updating processes and sustained attention (Bland and Schaefer, 2011).

Taken together, differences in P2 and LPC parameters show that high-level cognitive processes are engaged in both conditions of the UG, yet not at the same time during the processing of the stimulus.

### Behavioral Results

Finally, analyzing the behavioral results revealed a significantly higher loss for the participant in the proposer condition, indicating a higher risk whenever the participants were engaged in this condition. This supports our interpretation of a higher conflict in the proposer condition leading to the N2, as the proposer had to guess the other player’s response from the gains and losses experienced in previous trials (Oosterbeek et al., 2004).

### Limitations

A few limitations should be considered when interpreting these data.

First, source reconstruction is a method that is very sensitive to the placement of the electrodes, which should ideally be placed after MRI determination of the anatomy of each participant’s brain. As we did not have access to the individual MRIs of the participants, we used a standardized brain image for reconstruction, which is a method classically used in EEG studies. Moreover, the fact that we have a high density of electrodes adds to the reliability of the source reconstruction.

Second, the paradigm includes different response frequencies, offering nine response options in the proposer and only two in the responder condition. We therefore cannot totally exclude an influence on the ERP parameters. However, we suggest that this difference mostly affected the early N2 and P2 components, as previously mentioned in the discussion.

Finally, given the trend to a statistic difference of the offers between human participants and computer (*p* = 0.06), we cannot formally rule out qualitative differing strategies.

## Conclusion and Outlook

Our findings highlight a difference in the intensity of the engagement of neuronal systems, as well as a modified time-course during the social decision-making process, as shown by comparing both conditions of the UG. The presence of a supplementary component N2 only in the proposer condition points to an early conflict when choosing the amount to propose. However, the more pronounced FRN amplitude in the responder condition indicates that the conflict between accepting and refusing an offer occurs later in the responder. The modulation of the P2 indicates a smaller engagement of attentional processes in the responder condition. In contrast, changes in the LPC signal a higher involvement of motivated attention and updating processes in the responder condition.

The present results reveal a different functional dynamic in the same human participant according to its social position whilst playing the UG, even though the goal is identical in both conditions. Our ERP findings reveal bases of neural mechanisms involved in the theory of mind, which describes the cognitive ability to attribute different mental states to oneself and others. Characterizing ERPs of healthy participants acting as both proposer and responder thus provides valuable insight for the research on mentalizing processing steps. Subsequently, as deficits in this ability have been reported to occur in some psychiatric diseases (Baron-Cohen et al., 1985; Uekermann and Daum, 2008; Ng et al., 2015), using the UG coupled with EEG could help characterizing cognitive and social impairments in psychiatric patients.

## Author Contributions

PM and MCGM designed the study. SKH and GF acquired the data. SKH, PM, JR and GF analyzed the data. FRH and PM conducted the statistical analyses. AP critically evaluated the manuscript. All authors contributed to writing the article.

## Conflict of Interest Statement

The authors declare that the research was conducted in the absence of any commercial or financial relationships that could be construed as a potential conflict of interest.

## Footnotes

^1^http://www.marum.de/en/Leere_Seite_5.html#Section12094
